# Prediction of the future number of fall-related emergency medical services calls in older individuals

**DOI:** 10.1186/s12245-024-00654-w

**Published:** 2024-06-11

**Authors:** Shuji Uemura, Ryuichi Nakayama, Masayuki Koyama, Yukiko Taguchi, Naofumi Bunya, Keigo Sawamoto, Hirofumi Ohnishi, Eichi Narimatsu

**Affiliations:** 1 Department of Emergency Medicine, Sapporo Medical University School of Medicine, S-1, W-16, Chuo-ku, Sapporo, 060-8543 Japan; 2 Department of Emergency Medical Services, Life Flight and Disaster Medicine, Sapporo Medical University School of Medicine, S-1, W-16, Chuo-ku, Sapporo, 060-854356 Japan; 3 Department of Public Health, Sapporo Medical University School of Medicine, S-1, W-16, Chuo-ku, Sapporo, 060-854356 Japan; 4 Department of Nursing, School of Health Sciences, Sapporo Medical University, S S-1, W-16, Chuo-ku, Sapporo, 060-8556 Japan

**Keywords:** Emergency medical services, Falls, Future projection, Older population

## Abstract

**Background:**

Falls among older individuals contribute significantly to the rise in ambulance transport use. To recognize the importance of future countermeasures, we estimated the projected number and percentage of fall-related emergency medical service (EMS) calls.

**Methods:**

We examined the sex, age group, and location of falls among patients aged ≥ 65 years who contacted emergency services in Sapporo City from 2013 to 2021. Annual fall-related calls per population subgroup were calculated, and trends were analyzed. Four models were used to estimate the future number of fall-related calls from the 2025–2060 projected population: (1) based on the 2022 data, estimates from the 2013–2022 data using (2) Poisson progression, (3) neural network, (4) estimates from the 2013–2019 data using neural network. The number of all EMS calls was also determined using the same method to obtain the ratio of all EMS calls.

**Results:**

During 2013–2022, 70,262 fall-related calls were made for those aged ≥ 65 years. The rate was higher indoors among females and outdoor among males in most age groups and generally increased with age. After adjusting for age, the rate increased by year. Future estimates of the number of fall calls are approximately double the number in 2022 in 2040 and three times in 2060, with falls accounting for approximately 11% and 13% of all EMS calls in 2040 and 2060, respectively.

**Conclusion:**

The number of fall-related EMS calls among older people is expected to increase in the future, and the percentage of EMS calls will also increase; therefore, countermeasures are urgently needed.

**Supplementary Information:**

The online version contains supplementary material available at 10.1186/s12245-024-00654-w.

## Introduction

In Japan, the extended arrival time of ambulances at the scene due to increased emergency calls has become a social problem. Over the past 10 years, the average arrival time of an ambulance in Japan has increased by 2 min, which may delay the initiation of treatment for patients requiring urgent emergency medical service (EMS) care, such as cardiopulmonary arrest, hypoxia, and shock [1]. Older people aged ≥ 65 years account for 62.1% of all EMS transports, which means that approximately one out of every 10 older people is transported. Most of the increase in the number of EMS transports over the past 10 years has been due to the increase in EMS transports of older people [1]. The rise in the number of older people requiring ambulance transport is predominantly due to acute illness; however, the number of individuals in this age group requiring ambulance transport due to injuries has also increased by 1.5-fold [1]. As Japan’s population continues to age, it is anticipated that injuries among older individuals will also increase, potentially placing a burden on the emergency medical care system.

Falls among older individuals (falls in this study refer only to those not involving falling from a height) have become a global issue, with data from the United States of America for 2014 indicating that over one-fourth of older individuals experience a fall annually [2]. These falls result in increased morbidity, mortality, and healthcare costs [2–6]. In several countries, EMSs are working to prevent future falls through proactive interventions [7, 8]. In some regions of Japan, falls are reported to be the most common reason for ambulance transport for people aged ≥ 85 years and remain a significant challenge for ambulance transportation [9]. 

However, in Japan, EMSs do not consistently intervene to prevent falls, and limited measures are in place to reduce the number of cases requiring emergency transport for falls. It is unclear how many older individuals requiring ambulance transport due to falls will increase in the future.

This study aimed to calculate the projected number and percentage of future calls related to falls among older people to provide material to highlight the need for measures in pre-hospital care.

## Methods

This was a 10-year retrospective study using prehospital data from the Sapporo City Fire Bureau. This study was conducted in accordance with the principles of the Declaration of Helsinki and was approved by the Ethics Committee of Sapporo Medical University (approval number: 3-1-84) on April 27, 2022. The need for obtaining informed consent was waived due to the retrospective design of this study.

### Study setting

Sapporo is Japan’s fourth most highly populated city, with an area of 1,121.26 km [2] and a population of 1,938,331 in 2013 and 1,973,011 in 2022. The aging rate, based on people aged ≥ 65 years, in Sapporo City was 28.1% in 2022, which was roughly the same as the national average of 28.7%.[10]

In Japan, all emergency calls to the uniform emergency number 119 are handled by the dispatch center of the municipal fire department. Japan does not use a call triage system; hence, ambulances are dispatched to all calls, and all call dispatches are made with lights and sirens. Furthermore, since Japan does not have a non-transport protocol, all patients who do not decline will be transported to emergency medical institutions by ambulance.

### Outcomes

The primary outcome was the projected number of fall-related calls among older people. The secondary outcome was the proportion of falls among older people to the projected future number of emergency calls.

### Data collection

We reviewed all patient data for emergency calls in Sapporo City during the 10-year period from January 1, 2013, to December 31, 2022, using the Sapporo City Fire Department patient registry. Patients aged ≥ 65 years who were referred for ambulance transport due to a fall were eligible, except for those whose sex and age were unknown. We analyzed the number of emergency calls for each year by sex, age (in 5-year age groups), and whether the fall occurred indoors or outdoors. Indoor falls were defined as falls in roofed areas, such as the home, workplace, older adult care facility, or public facility. Outdoor falls were defined as falls outside or on the premises of a building but without a roof and were checked at the discretion of each EMT.

The sex and age (in 5-year age groups) of Sapporo City’s population are based on the figures from the Basic Resident Register of the Sapporo City Hall Database as of July 1 of each year [11]. We used future population projections of Sapporo City between 2025 and 2060, as estimated by Sapporo City Hall, to estimate the number of fall-related calls in the future [12]. (Supplementary Table 1). The cohort factor method was used with the population by sex in the 5-year age group from the Ministry of Internal Affairs and Communications Statistics Bureau “Population Census” as the reference population. The annual changes that occur with aging in the age-specific population utilized factors such as fertility rate, sex ratio at birth, survival rate, and net migration rate.

### Data analysis

The annual fall-related call rate by age group (5-year age groups) in those ≥ 65 years old was calculated by sex by dividing the number of fall-related calls by the population of each age and sex subgroup. The same calculations were performed separately for indoor and outdoor occurrences. The difference in rates of fall-related calls between males and females was tested using Student’s *t*-test.

Age standardization was performed by the direct method using the average age distribution of 5-year-old male and female groups in Sapporo City from 2013 to 2022 as the reference population. At age ≥ 65 years, linear regression analysis was performed on the bivariate relationship between the number of fall-related calls per 100,000 population and annual trends by sex. To consider the influence of the COVID-19 pandemic, we divided the analysis into two periods, 2013–2019 and 2013–2022 [13]. 

Four calculation methods were used to obtain future estimates from 2025 to 2060. The first is the call rates by sex and age group in 2022, which were multiplied by the respective estimated future population and summed. The second used trends identified from 2013 to 2022 to make projections for the future. Poisson regression models, including sex, age group, and calendar year, were used [13]. Using the maximum likelihood method, the Akaike Information Criterion (AIC) was used as a measure of the predictive ability. The third method employed machine learning using the neural network model. Neural networks can model non-linear relationships in data, making them particularly useful for complex forecasting tasks. Specifically, we used the 2013–2022 data, with sex, age group, and calendar year as explanatory variables. Nested cross-validation was performed to prevent machine learning overfitting as much as possible. For cross-validation, the data were divided into four nested groups. The fourth group was used as the test set, and the remaining data were further divided into five equal groups. One subgroup of the internal folds was treated as the validation set, and the model was trained on the remaining data. In total, 4 × 5 models were fitted (K = 4, L = 5). The fourth was done the same way as the third but with a model using the years 2013–2019, which is unaffected by the COVID-19 pandemic. The number of emergency calls for each year was obtained by fitting the predictive models to the predicted population in age group, by sex, from 2025 to 2060 and summing the results. Each future estimate was calculated for falls-related calls and all emergency calls, respectively, as well as for the percentage of falls-related calls in all EMS calls. Falls-related calls were calculated separately for indoor and outdoor calls.

Analyses were performed using JMP software (JMP Pro 16 for Windows, SAS Institute, Cary, NC, USA).

## Results

### Incidence rates

**Fig. 1:**
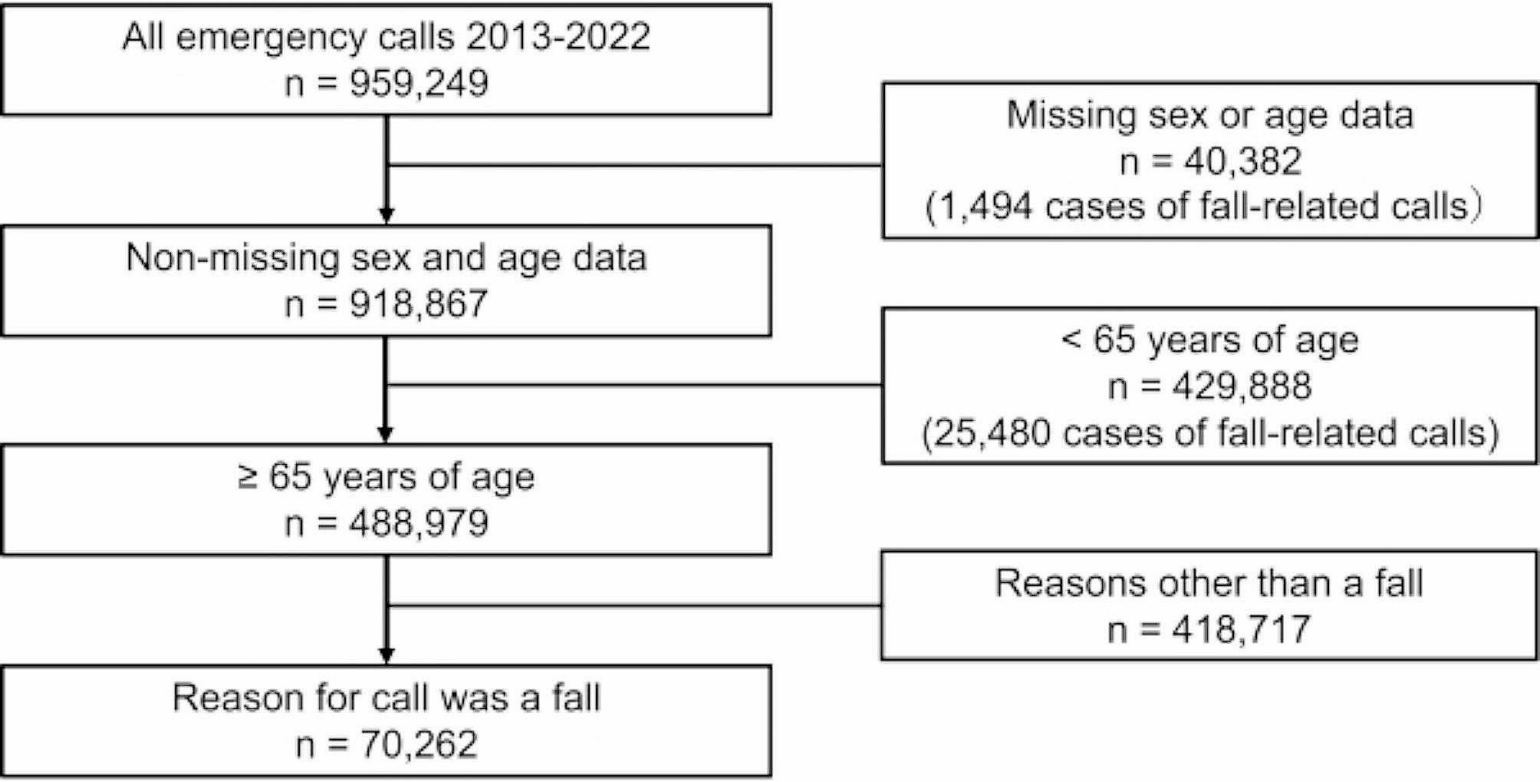
Study subject selection flow chart using the Sapporo City Fire Department patient registry

During 2013–2022, 70,262 fall-related calls were made for people aged ≥ 65 years, with an annual average of 7,026. Overall, 63% of patients were female, and 64% of falls occurred indoors. The number of fall-related calls per 100,000 population aged ≥ 65 years was 1,391(95% confidence interval [CI] 1,305–1,478) (Fig. 1, Supplement Table 1).

**Table 1:** Estimated number of future fall-related calls obtained using the four models for 2025–2060 and their ratio to the actual number in 2022

	2025	2030	2035	2040	2045	2050	2055	2060
Estimated using the 2022 data	10,378	11,589	12,871	13,804	14,154	14,447	14,731	15,280
Compared with 2022	1.1	1.2	1.4	1.5	1.5	1.6	1.6	1.6
Estimated using Poisson progression	10,343	12,518	15,082	17,737	19,887	22,084	24,466	27,584
95% confidence intervals	10,164–10,525	12,199–12,847	14,566–15,617	16,973–18,537	18,854–20,979	20,742–23,516	22,768–26,296	25,436–29,920
Compared with 2022	1.1	1.3	1.6	1.9	2.1	2.4	2.6	3.0
Estimated using neural boost based on the data through 2019	10,323	12,732	15,548	18,250	20,375	22,309	23,827	25,263
Compared with 2022	1.1	1.4	1.7	2.0	2.2	2.4	2.6	2.7
Estimated using neural boos	11,181	17,465	22,854	26,246	28,680	31,019	32,648	34,043
Compared with 2022	1.2	1.9	2.5	2.8	3.1	3.3	3.5	3.7

**Fig. 2:**
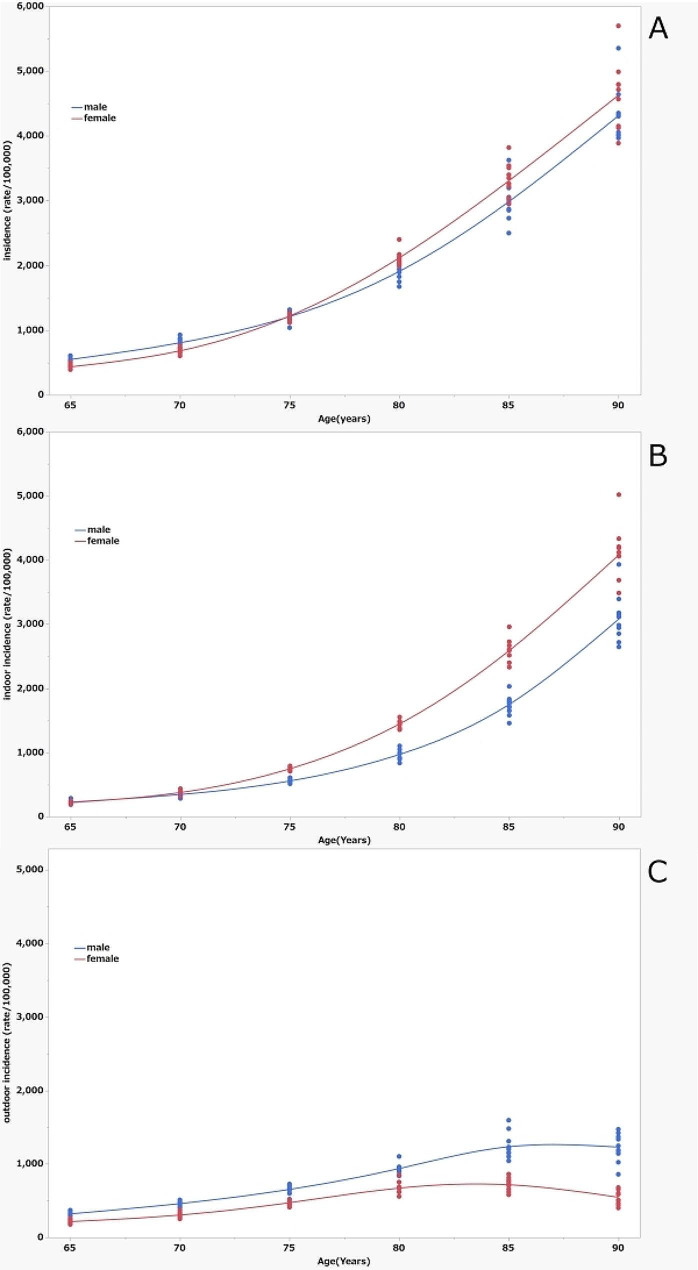
Fall-related calls (rate/100,000 person-years) in Sapporo, by age and sex. (**a**) Rates for all falls, (**b**) rates for indoor falls, (**c**) rates for outdoor falls. Asterisk marks (*) indicate significantly higher rates in males than in females. Cross marks (+) indicate significantly higher rates in females than in males

Figure 2a shows a plot of fall-related call rates for each year by age and sex. The age-specific incidence rates in both males and females increased with increasing age subgroups. The fall-related call rate was significantly higher in the male subgroups of 65–69 (*p* < 0.0001) and 70–74 years (*p* < 0.0001) and in the female subgroups of 80–84 (*p* = 0.0009) and 85–89 (*p* = 0.0210) than in the other sexes. Figure 2b shows the rate of indoor fall-related calls, which was significantly higher in the female subgroups of 75–79, 80–84, 85–89, and 90 and above (all *p* < 0.0001) than for males and increased exponentially with age. Figure 2c shows the rate of outdoor fall-related calls. The rate was higher in males of all age groups (all *p* < 0.0001) than in females and increased up to the age 85 subgroup but then showed a decreasing trend in the age group of ≥ 90 years. By subgroup, the highest value was 4,087 per 100,000 (95% CI 3782–4393) calls for indoor falls for females aged ≥ 90 years, while the lowest rate was 216 per 100,000 (95% CI 198–235) for outdoor falls among females aged 65–70. The highest and lowest call rates had an approximate 20-fold difference.

### Time trends

From 2013 to 2019, which were unaffected by the COVID-19 pandemic, annual trends in fall-related call rates correlated well with an R-squared of 0.87 for females and 0.84 for males and increased significantly for both females and males even after age adjustment (Table 2). As the trend became smaller when age adjustment was performed, age had a greater influence on fall-related call rates than did the year. In the analysis of the 2013–2022 data, there was a linearity in age-adjusted values for females but not for males, which was considered to be related to the influence of the COVID-19 pandemic.

**Table 2:** Linear regression analysis of the bivariate relationship between the number of fall-related calls per 100,000 population aged ≥ 65 years and annual trends by sex, with and without age adjustment, performed for the 2013–2019 and 2013–2022 data

			Intercept	β	Trend *p* value	*R* square
2013–2019	Unadjusted	Female	-77466.2	39.1434	0.0018	0.87906
		Male	-41751.9	21.3087	0.0034	0.84502
	Age-adjusted	Female	-51,714	26.3795	0.0073	0.79142
		Male	-27357.7	14.1758	0.0289	0.64828
2013–2022	Unadjusted	Female	-86587.9	43.6663	0.0006	0.79446
		Male	-38595.4	19.7385	0.0440	0.41621
	Age-adjusted	Female	-47747.0	24.4135	0.0076	0.61056
		Male	-13800.9	7.4489	0.3736	0.09991

### Predicted number of fall-related calls

**Fig. 3:**
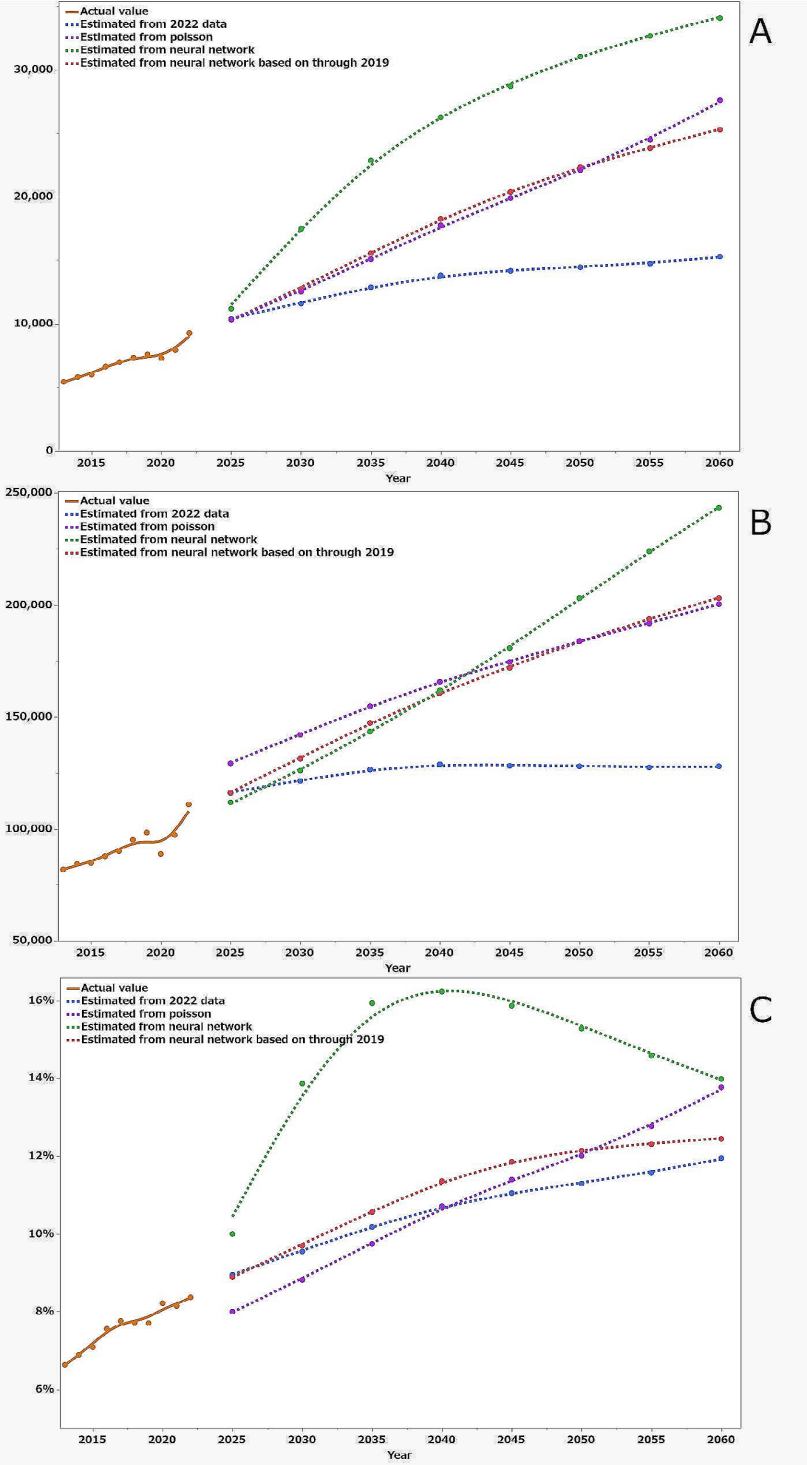
The solid line represents the actual data from 2013 to 2022. The dashed line shows the estimated future number of fall-related calls from the projected population for 2025–2060 obtained using four models. The blue dashed line is an estimate based on the 2022 data, the purple dashed line is an estimate from the 2013–2022 data obtained using the Poisson progression method, the green dashed line is estimate from the 2013–2022 data obtained using neural networks, and the red dashed line is from the 2013–2019 data obtained using neural networks. (**a**) Number of fall-related calls, (**b**) number of all EMS calls, and (**c**) percentage of fall-related calls to total calls

Figure 3a shows the projected number of future fall-related calls from 2025 to 2060 based on the above four forecast models. The goodness of fit of the Poisson regression model was AIC = 2375 indoors and AIC = 4459 outdoors. Neural network model correlations using the 2013–2022 data had R-square and root mean square error (RMSE) values of 0.98 and 157 for indoor falls and 0.91 and 0.96 for outdoor falls, respectively. Neural network model correlations using the 2013–2019 data had R-square and RMSE values of 0.99 and 124 for indoor falls and 0.90 and 97 for outdoor falls, respectively. The population of Sapporo City aged ≥ 65 years will peak in 2045, by reaching 1.24 times the population in 2022. Conversely, the predicted number of fall-related calls from people aged ≥ 65 years will not peak in any model equation, ranging from 11,589 to 17,465 in 2030, 13,804 to 26,245 in 2040, and 15,280 to 34,043 in 2060. Compared with the actual number of 9,277 in 2022, these predicted values are 1.2–1.9 in 2030, 1.5–2.8 in 2040, and 1.6–3.7 times higher in 2060, respectively (Table 1). Figure 3b shows the projected number of future calls for the above four forecast models. The goodness of fit of the Poisson regression model was AIC = 522,692. Neural network model correlations using data from 2013 to 2022 had R-square and Root mean square error (RMSE) values of 0.97 and 1093. Neural network model correlations using the 2013–2019 data had R-square and RMSE values of 0.98 and 949. The result for the number of all emergency calls estimated based on 2022 data was the smallest of the four estimates, peaking in 2040 and declining thereafter. The percentage of fall-related calls among all EMS calls exceeded 10% in 2035 and increased to 11.9% in 2060. Poisson analysis and neural network estimates through 2019 were similar and larger than those estimated based on the 2022 data. Neural network estimates for data through 2022 showed the greatest increase, with the number of fall-related calls increasing initially and then smoothing out, whereas the number of total EMS calls increased linearly. Hence, the percentage increased significantly through 2040 and then decreased. As a result, the percentage of fall-related call estimates for older people in all calls estimates ranged from 8.8 to 13.9% in 2030, 10.7 to 16.2% in 2040, and 11.9 to 14% in 2060.

## Discussion

This is an important study for considering the future EMS system for an aging society, which estimates the future increase in the number of fall-related EMS calls due to the aging population. This study found that, although the number of fall-related calls for the population increases with increasing age, the trend and the proportion of calls per age group made for males and females differ based on whether the fall occurred indoors or outdoors and that the number of fall-related calls tends to increase with each year after adjustment for age. We used four models. The first model is based on the 2022 data, applied only the variables of age and sex and does not consider the actual increase in population that may occur each year. It is considered to be a minimum figure. Conversely, neural networks are algorithms that can be used to perform nonlinear statistical modeling, providing a new alternative to regression analysis [14]. However, the explosive increase in the number of fall-related calls in the neural network using data through 2022 is due to the COVID-19 pandemic, where the number of cases was low in 2020 and 2021 and returned to normal in 2022; however, the slope possibly was considered directly into the calculation. Estimates from Poisson regression have been used in previous studies to estimate future hip fractures [13]. Neural networks using data through 2019, which were similar to those of the results of the poisson regression, can be trusted because they are based on reliable and stable data from 2013 to 2019. Based on the above, if no measures are taken, the fall-related call rate will be approximately two times higher in 2040 and three times higher in 2060, with falls accounting for approximately 11% and 13% of all EMS calls in 2040 and 2060; we believe this is reasonable. Fall-related EMS calls were expected to continue to grow at least through 2060, although the population of older people will peak in 2045. This was thought to be because a large number of indoor falls occur in super-aged people.

A 2017 study using a U.S. Nationwide Emergency Department Sample data examined the incidence of falls in patients aged ≥ 65 years relative to the population by age group, divided into indoor and outdoor, and found, similar to this study, that the incidence increased with increasing age, with indoor incidence being higher than outdoor incidence [15]. Comparing the incidence rate per 100,000 population, the number of emergency department visits in the U.S. was 12,176, and the number of EMS calls in Japan was 1,681, with a difference of 7.2 times. This may be because the U.S. data includes patients who went directly to the emergency department without an ambulance. Data using EMS data in Tokyo in 2005 reported that the rate was 3,850/100,000 population, which was 3.55 times higher than the 1,140/100,000 population rate among 65–84-year-olds, similar to our results [9]. Many older individuals who attend community classes, such as fall prevention classes, are relatively young. Therefore, a more aggressive preventive approach should be considered for super-aged people.

Furthermore, this study found that, excluding the impact of COVID-19, the annual rate of fall-related calls tended to increase, even after adjusting for age. This indicates the need for further fall prevention measures to reduce the fall-related call rate. In the United Kingdom, EMSs have adopted a system in which fall patients who call for an ambulance are evaluated and referred to a fall service, which has successfully reduced the number of fall-related calls [16]. In the United States, community paramedics visited homes to assess the risk of falls, including for individuals and the home environment, for preventive intervention, and have reportedly reduced the rate of fall-related calls [8]. Conversely, in Japan, EMSs have not yet intervened to prevent falls, but there is a need to consider such interventions.

Japan may also need to consider introducing a non-transport protocol to deal with the exponentially increasing number of fall-related calls. A quarter of older individuals who have experienced falls are not taken to the emergency department after being evaluated and are treated by paramedics [17, 18]. Several countries have introduced non-transport protocols, including for falls among older individuals, and their safety has been verified [19, 20]. To introduce a non-transport protocol in Japan, public consensus and education of paramedics will be necessary.

Our study had some limitations. First, we added only four factors to our future estimates: age, sex, calendar year, and indoor or outdoor location of occurrence of falls, and did not consider the relationships among the other factors. Second, nested cross-validation was performed to prevent machine learning overfitting as much as possible, but accuracy is limited. Third, the figures for 2020 and 2021 were affected by the COVID-19 pandemic. Fourth, the current emergency system may not be financially viable due to changes in Japan’s socioeconomic circumstances.

## Conclusion

In conclusion, if the current situation continues, the number of the fall-related call for older people in Sapporo will be 1.9 to 2.0 times higher in 2040 and 2.7 to 3.0 times higher in 2060, and the future estimates show that the ratio of falls to EMS calls will increase to 10.7 to 11.4% in 2040 and 12.4 to 13.8% in 2060. Fall-related EMS calls are expected to continue to grow at least through 2060, even though the population growth of older people will peak in 2045. Hence, preventive measures should be considered for these data in particular. It may also be necessary to consider repeating these studies in other cities to get a more general idea of the interventions needed in Japanese cities.

### Electronic supplementary material

Below is the link to the electronic supplementary material.

Supplementary Material 1

## Data Availability

Some data is derived from public domain sources (with links). Other data is incorporated into the article and its online supplements.
