# Supplementary material for: Prediction of the future number of fall-related emergency medical services calls in older individuals
Source: Int J Emerg Med. 2024 Jun 11;17:72. doi: 10.1186/s12245-024-00654-w (PMC11165859; doi:10.1186/s12245-024-00654-w)
Supplement: Supplementary file 1 — Supplementary Material 1 [file 12245_2024_654_MOESM1_ESM.docx]

Supplement Table 1. Projected future population aged 65 years and over in Sapporo City from 2025 to 2060 estimated by Sapporo City

| sex | Age | 2025 | 2030 | 2035 | 2040 | 2045 | 2050 | 2055 | 2060 |
| --- | --- | --- | --- | --- | --- | --- | --- | --- | --- |
| female | 65-69 | 64000 | 68000 | 72000 | 81000 | 72000 | 64000 | 57000 | 55000 |
| female | 70-74 | 71000 | 62000 | 67000 | 71000 | 79000 | 71000 | 63000 | 56000 |
| female | 75-79 | 75000 | 68000 | 59000 | 64000 | 68000 | 77000 | 68000 | 61000 |
| female | 80-84 | 54000 | 70000 | 64000 | 56000 | 61000 | 65000 | 73000 | 65000 |
| female | 85-89 | 41000 | 47000 | 62000 | 56000 | 50000 | 54000 | 58000 | 67000 |
| female | 90- | 36000 | 42000 | 50000 | 65000 | 71000 | 68000 | 69000 | 75000 |
| male | 65-69 | 56000 | 59000 | 63000 | 73000 | 65000 | 58000 | 53000 | 51000 |
| male | 70-74 | 60000 | 52000 | 55000 | 59000 | 69000 | 62000 | 55000 | 51000 |
| male | 75-79 | 57000 | 53000 | 47000 | 49000 | 53000 | 62000 | 56000 | 50000 |
| male | 80-84 | 34000 | 47000 | 44000 | 39000 | 41000 | 45000 | 53000 | 48000 |
| male | 85-89 | 20000 | 24000 | 33000 | 32000 | 28000 | 31000 | 34000 | 40000 |
| male | 90- | 12000 | 14000 | 18000 | 25000 | 26000 | 26000 | 26000 | 30000 |

Supplement Table 2. Characteristics of fall-related calls for emergency services by year among older individuals and the population of Sapporo City aged 65 and over
